# Books Reviewed

**Published:** 1863-03

**Authors:** 


### BOOKS REVIEWED.

*The Principles and Practice of Obstetrics. By* GUNNING S. BEDFORD, M. D., *Professor of Obstetrics, the Diseases of Women and Children, and Clinical Obstetrics, in the University of New York; author of "Clinical Lectures on the Diseases of Women and Children." Illustrated by four Colored Lithographic Plates and ninety-nine wood engravings. Third Edition, carefully revised and enlarged. New York:* WILLIAM WOOD & CO., 61 *Walker street.*

Gunning S. Bedford's work on Obstetrics has passed to a third edition, and a new chapter been added on *Phlegmasia Dolens,* besides additions to the text throughout. It has been carefully revised and enlarged, and as it now appears has increased claims upon the profession. This book has been adopted as the Text Book on Obstetrics in fourteen of our medical schools, and will soon be adopted by all in this country. Its popularity abroad is equally as great as at home, as shown by its translation into French and German, and by the favorable notices of the foreign journals. We feel a pride in this unparallelled success of an American author; it speaks volumes for the book, since the French and Germans are sufficiently slow in awarding great merit to any foreign author.

Why is it, that this book has obtained so great popularity, and in so short time come to be regarded as indispensable to every practicing physician? The answer to this question becomes apparent upon perusal; on every page may be seen reasons why physicians in obstetrical practice should possess it as a daily guide and reference. It contains such plain practical teaching, is so philosophical in argument, so rational in theory, careful in investigation, and so recent and scientific in the treatment of all the various diseases of which it treats, that physicians cannot neglect its acquaintance. The obscure, deep, and uncertain, as well as the plain, common and established facts and theories are fully considered; no department of obstetrical practice has been neglected, each subject has been fairly treated without bias, or desire to establish or destroy pre-conceived theory or opinion. Previous authors are respected, and their views and opinions are not disregarded, while the governing object in the whole work, is plainly, to establish and teach what is true, and to show clearly whatever is regarded as false.

The style of this work is highly worthy of mention; it is original, peculiar to the author, and highly attractive, imparting an interest which the subjects could otherwise hardly be supposed to possess. In a word, it is the "Book of Books," in obstetrics, and should be the daily companion of the Obstetrician.

